# Preoperative eGFR, age, and sex predict early postoperative renal changes in acromegaly

**DOI:** 10.1210/jendso/bvag126

**Published:** 2026-05-31

**Authors:** Keiko Tomiyama, Izumi Fukuda, Mikiko Okazaki-Hada, Shunsuke Kobayashi, Mototsugu Nagao, Shigeyuki Tahara, Masato Iwabu

**Affiliations:** Department of Endocrinology, Metabolism, and Nephrology, Graduate School of Medicine, Nippon Medical School, Tokyo 113-8603, Japan; Department of Endocrinology, Metabolism, and Nephrology, Graduate School of Medicine, Nippon Medical School, Tokyo 113-8603, Japan; Department of Endocrinology, Metabolism, and Nephrology, Graduate School of Medicine, Nippon Medical School, Tokyo 113-8603, Japan; Department of Endocrinology, Metabolism, and Nephrology, Graduate School of Medicine, Nippon Medical School, Tokyo 113-8603, Japan; Department of Endocrinology, Metabolism, and Nephrology, Graduate School of Medicine, Nippon Medical School, Tokyo 113-8603, Japan; Division of Diabetology and Metabolism, Department of Internal Medicine, Tokyo Women's Medical University School of Medicine, Tokyo 162-8666, Japan; Department of Neurological Surgery, Nippon Medical School Musashikosugi Hospital, Kawasaki, Kanagawa 211-8533, Japan; Department of Endocrinology, Metabolism, and Nephrology, Graduate School of Medicine, Nippon Medical School, Tokyo 113-8603, Japan

**Keywords:** acromegaly, postoperative change, renal function, sex-based differences

## Abstract

**Objective:**

Acromegaly induces renal alterations. However, early postoperative renal changes following rapid hormonal correction remain unclear. We assessed early postoperative changes in estimated glomerular filtration rate (eGFR) and their associated factors, focusing on preoperative eGFR, age, and sex.

**Methods:**

We retrospectively analyzed 44 patients with acromegaly who underwent trans-sphenoidal surgery between 2012 and 2024. We analyzed renal function using preoperative eGFR and changes in eGFR at postoperative day 14 (POD14) relative to preoperative values (ΔeGFR). Patients were stratified by preoperative eGFR (≥ 90 vs <90 mL/min/1.73 m^2^), age (<65 vs ≥65 years), and sex. Multivariate regression analyses were performed to identify factors associated with preoperative eGFR and postoperative changes.

**Results:**

The eGFR had decreased significantly by POD14, especially among patients with higher preoperative eGFR (ΔeGFR: −27, *P* < .0001) and in females. Significant declines in eGFR occurred in both younger and older patients, with greater reductions in younger patients. Among older patients, 3 exhibited declines in eGFR to <60 mL/min/1.73 m^2^ at POD14. Multivariate analysis revealed an association between female sex and greater reductions in IGF-1 standard deviation scores with larger ΔeGFR.

**Conclusion:**

To our knowledge, this is the first study to demonstrate that rapid postoperative hormonal reductions in acromegaly are associated with early eGFR declines, particularly in patients with higher baseline eGFR, females, and younger patients. While eGFR declines are modest, patients with advanced age and metabolic comorbidities may be at risk of clinically relevant renal impairment and require careful monitoring.

The most common cause of acromegaly is due to growth hormone (GH)-secreting pituitary neuroendocrine tumors [1]. Acromegaly induces chronic GH hypersecretion, resulting in elevated circulating and tissue levels of insulin-like growth factor-1 (IGF-1) [1]. GH exerts direct effects via the GH receptor (GHR)/Janus kinase 2/signal transducer and activator of transcription 5 and extracellular signal-regulated kinase1/2 pathways, as well as indirect effects through circulating or paracrine IGF-1, which activates the IGF-1 receptor (IGF-1R).

Both GHR and IGF-1R are expressed in the kidneys, indicating that the kidneys are GH and IGF-1 target organs. Consequently, both functional and morphological renal alterations are observed in acromegaly [2]. Previous studies have reported that active acromegaly induces glomerular hyperfiltration and a consequent decrease in the estimated glomerular filtration rate (eGFR) following treatment [2, 3]. This decline is considered to reflect a physiological adjustment, rather than kidney injury [2, 3]. To date, this postoperative eGFR decline has been confirmed within 3-6 months after the administration of effective treatments for acromegaly [4, 5]. Fujio et al [5] reported that eGFR normalization occurred predominantly in patients with preoperative hyperfiltration (eGFR ≥ 90 mL/min/1.73 m^2^) at 3 months postoperatively, whereas those with lower baseline eGFR values experienced minimal changes.

The early postoperative period is generally characterized by unstable hemodynamics and an increased risk of acute kidney injury (AKI) [6]. In general population, older age and reduced renal reserve are known as risk factors for AKI [7], and female sex has been reported to be protective against hospital-associated AKI, suggesting potential sex-based differences in renal susceptibility [8].

However, to our knowledge, no studies to date have investigated early postoperative renal changes following acromegaly treatment with particular consideration for age and sex. Therefore, we aimed to evaluate early postoperative renal function following trans-sphenoidal surgery in patients with acromegaly and identify the clinical factors associated with postoperative eGFR changes, including variables such as preoperative eGFR, age, and sex.

## Subjects and methods

### Patients

This retrospective study included 56 patients with acromegaly who underwent trans-sphenoidal surgery at Nippon Medical School Hospital between 2012 and 2024. To specifically evaluate renal function changes associated with the correction of GH/IGF-1 excess, this study focused on patients who achieved remission after surgery. The remission was defined based on IGF-1 normalization, as described in Methods. Nine patients were excluded for the following reasons: GH/thyrotropin cosecreting tumor (n = 1), pituitary apoplexy (n = 2), preoperative medical therapy (n = 2), stroke (n = 1), high-dose steroid use (n = 1), and missing data (n = 2). Among the remaining 47 patients, 3 patients were excluded postoperatively because they required additional therapy to treat persistent disease activity. Consequently, a total of 44 patients who achieved remission were included in the final analysis.

### Methods

Clinical and biochemical parameters were assessed at predefined time points, including preoperatively and on postoperative day 14 (POD14). Data from 3 months postoperatively and during subsequent follow-up visits were analyzed when available.

IGF-1 normalization was defined as an IGF-1 level below 2 standard deviation scores (SDS), based on age- and sex-adjusted reference ranges [9], corresponding to values within the upper limit of normal. Biochemical remission in acromegaly is conventionally assessed at least 12 weeks after surgery, as recommended by recent clinical guidelines [10]. However, delayed remission has also been reported, with some patients achieving normalization of IGF-1 levels during longer-term follow-up after initial surgery [11]. Therefore, patients who achieved IGF-1 normalization without additional therapy during follow-up were considered to have achieved remission and included in our analysis.

Renal function was assessed using eGFR (mL/min/1.73 m^2^), which was calculated using the revised Japanese formula that incorporates age and serum creatinine (SCr) level: eGFR (mL/min/1.73 m^2^) = 194 × age^−0.287^ × SCr^−1.094^ (if female, ×0.739). The difference between POD14 and preoperative eGFR (eGFR at POD14—preoperative eGFR) was defined as ΔeGFR. Similarly, ΔGH, ΔIGF-1, ΔIGF-1 SDS, and Δcreatine kinase (ΔCK) were defined as the difference between POD14 and preoperative value. As an additional exploratory analysis, percentage changes in eGFR, GH, and IGF-1 were calculated to assess within-patient relative changes from baseline. Percentage change was defined as (postoperative value − preoperative value)/preoperative value × 100. We selected POD14 as it represents the earliest stable postoperative time point at our institution when hydration, steroid tapering, and hemodynamics typically normalize.

To evaluate early postoperative renal changes, patients were stratified by the timing of hormonal normalization (IGF-1 < 2.0 SDS at POD14 vs >3 months). Based on previous evidence suggesting that postoperative eGFR decline is limited to patients with preoperative eGFR values of ≥90 mL/min/1.73 m^2^ [5], our patients were subsequently stratified by baseline renal function (≥90 vs <90 mL/min/1.73 m^2^). Multivariate linear regression analysis was performed using preoperative eGFR as the dependent variable to identify which clinical factors were associated with baseline renal function. Significant variables were then used for subsequent stratified analyses.

To identify the factors associated with postoperative renal function change, a multivariate regression analysis was performed using ΔeGFR as the dependent variable. The covariates included age, sex, body mass index (BMI), ΔGH, ΔIGF-1 SDS and the use of renin–angiotensin system inhibitors (RASi), including angiotensin-converting enzyme inhibitors or angiotensin receptor blockers, or diuretics. None of the patients were receiving medications known to affect renal hemodynamics, including nonsteroidal anti-inflammatory drugs or sodium–glucose cotransporter 2 inhibitors, at any point during the study.

The clinical variables included GH, IGF-1, IGF-1 SDS, their respective postoperative changes (ΔGH, ΔIGF-1, and ΔIGF-1 SDS), sex, BMI, fasting blood glucose level, glycated hemoglobin level, comorbidities (diabetes mellitus and hypertension), and the use of RASi or diuretics. CK were assessed to support the interpretation of creatinine-based eGFR. Because sCr is influenced by muscle mass [12], postoperative changes in muscle mass may bias eGFR estimation. Previous studies have also reported a positive association between CK levels and skeletal muscle mass [13]. Therefore, CK was analyzed as a muscle-derived enzyme to provide supportive information on muscle-related changes.

### Statistical analysis

Continuous variables are expressed as medians and interquartile ranges. The distribution of continuous variables was assessed using the Shapiro–Wilk test to determine normality. Comparisons of preoperative vs POD14 values for ΔeGFR, ΔGH, ΔIGF-1, and ΔIGF-1 SDS were performed using the Wilcoxon signed-rank test. For analyses involving repeated measurements at 3 or more time points (eg, preoperatively and at 14 days, 3 months, 1 year, and 3 years postoperatively), we applied the Friedman test, followed by post hoc Wilcoxon signed-rank tests with Bonferroni correction. Comparisons of ΔCK between independent groups (male vs female, < 65 vs ≥65 years) were conducted using the Mann–Whitney U test.

Correlations between variables were assessed using Spearman's rank correlation coefficients. Age- and sex-adjusted associations were evaluated using Spearman correlation analyses based on residuals from linear regression models. Multivariate linear regression analyses were performed to identify the independent predictors of renal function. First, a model using preoperative eGFR as the dependent variable was constructed to identify factors associated with baseline renal function. This model included age, sex, BMI, preoperative GH, IGF-1 SDS, and the use of RASi or diuretics as covariates. A second model using ΔeGFR as the dependent variable included age, sex, BMI, ΔGH, ΔIGF-1 SDS, and the use of RASi or diuretics as covariates. Because ΔIGF-1 and ΔIGF-1 SDS were highly correlated, ΔIGF-1 were not included in the same model to avoid multicollinearity; ΔIGF-1 SDS was retained as an age- and sex-adjusted measure of hormonal change.

As an additional exploratory analysis, percentage changes in eGFR, GH, and IGF-1 were calculated as (postoperative value − preoperative value)/preoperative value × 100 to assess relative changes within patients. Multivariate regression analyses using %eGFR as the dependent variable included %GH and %IGF-1, along with age, sex, BMI, and the use of RASi or diuretics. Because %IGF-1 and %IGF-1 SDS were highly correlated, %IGF-1 SDS was not included in the same model. %IGF-1 was selected to directly represent relative changes in IGF-1 levels. Any two-sided *P* values of <.05 was considered statistically significant. All statistical analyses were performed using GraphPad Prism version 10.4.2 (GraphPad Software, San Diego, CA, USA) and JMP Pro version 18 (SAS Institute, Cary, NC, USA).

### Ethical considerations

This study was approved by the Ethics Committee of Nippon Medical School Hospital (reference no. B-2021-491), and was conducted in accordance with the principles of the Declaration of Helsinki. We obtained patient data retrospectively and disclosed the present study information, giving participants an opportunity to opt out in accordance with the guidelines of Nippon Medical School and its Faculty of Medicine Ethics Committee. The data were routinely obtained, and were essential for the appropriate management of pituitary disease. The requirement for informed consent was waived because this study was retrospective in design. All data were anonymized to protect patient privacy.

## Results

### Patient characteristics

A total of 44 patients with acromegaly were included in the final analysis. Their baseline characteristics are presented in Table 1. The median age was 50 years, and 25 (57%) were females. The median preoperative eGFR was 109 mL/min/1.73 m^2^. Of the total patient cohort, 33 had preoperative eGFR of ≥90 mL/min/1.73 m^2^, 9 had values of 60-89 mL/min/1.73 m^2^, and 2 had values of <60 mL/min/1.73 m^2^.

**Table 1:** Comparison of the baseline characteristics of our cohort of patients with acromegaly

	All	Age < 65 years	Age ≥ 65 years	*P* value
N	44	34	10	
Age (years)	50 (41-62)	45 (40-52)	69 (65-71)	<.0001*^a^*
Sex (male/female)	19/25	14/20	5/5	
BMI (kg/m^2^)	24.8 (22.4-26.5)	24.9 (22.9-26.5)	24.1 (21.2-29.1)	.67*^a^*
GH (ng/mL)	12.07 (6.96-31.88)	13.53 (6.67-33.54)	8.50 (6.59-25.17)	.48*^a^*
IGF-1 (ng/mL)	508 (400-703)	525 (436-696)	440 (378-736)	.46*^a^*
IGF-1 SDS	6.1 (5.4-8.1)	6.1 (5.3-8.2)	5.9 (5.3-8.3)	.94*^a^*
Serum creatinine (mg/dL)	.54 (0.41-0.68)	0.51 (0.41-0.65)	0.62 (0.52-0.73)	.11*^a^*
eGFR (mL/min/1.73 m^2^)	109 (89-129)	120 (100-129)	80 (62-106)	.01*^a^*
≥90 mL/min/1.73 m^2^, n (%)	33 (75)	29 (85)	4 (40)	
60-89 mL/min/1.73 m^2^, n (%)	9 (20)	4 (12)	5 (50)	
<60 mL/min/1.73 m^2^, n (%)	2 (5)	1 (3)	1 (10)	
CK (U/L)	92 (67-119)	87 (66-125)	98 (81-114)	.47*^a^*
Diabetes mellitus, n (%)	8 (18)	2 (11)	6 (60)	<.0001*^b^*
RASi, n (%)	12 (27)	5 (14)	7 (70)	< .001*^b^*
Diuretic, n (%)	5 (11)	3 (8)	2 (20)	.33*^b^*

Data are shown as medians and interquartile ranges, with binary data as numbers and percentages.

Abbreviations: BMI, body mass index; CK, creatine kinase; eGFR, estimated glomerular filtration rate; GH, growth hormone; IGF-1, insulin-like growth factor-1; RASi, renin–angiotensin system inhibitors; SDS, standard deviation score.

^*a*^Mann–Whitney *U* test

^*b*^χ^2^ test (compared between age <65 vs ≥65 years).

### Early postoperative eGFR changes according to hormonal normalization

Of the 44 patients, 25 achieved IGF-1 < 2.0 SDS by POD14, while 19 required more than 3 months to normalize. Among these 19 patients, 12 had eGFR data available at 3 months and were therefore included in the longitudinal analysis. Although not all patients achieved IGF-1 normalization by 3 months, normalization was confirmed in all cases during subsequent follow-up without the need for additional therapy.

**Figure 1:**
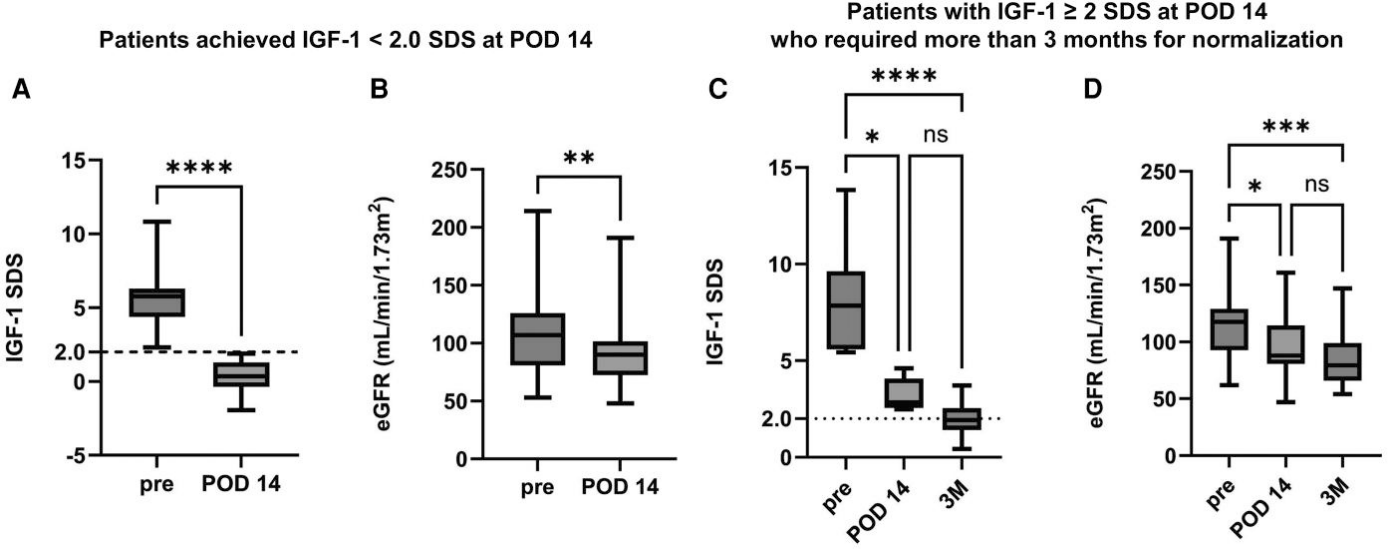
Early postoperative eGFR changes according to the timing of IGF-1 normalization. (A, B) Patients whose IGF-1 values normalized by postoperative day 14 (POD14) (n = 25) showed a significant decline in eGFR from baseline. (C, D) Patients requiring >3 months for IGF-1 normalization (n = 19, of whom 12 had eGFR data available at 3 months postoperatively) also showed a decline by POD14, with no further significant change at 3 months. Data are presented as medians and interquartile ranges. Data are shown as medians ± interquartile ranges. *P < .05; **P  *<* .01; ***P  *<* .001; ****P  *<* .0001; ns, not significant.

When stratified by IGF-1 level at POD14, the patients with IGF-1 < 2.0 SDS at POD14 (n = 25) showed a significant median eGFR decrease, from 107 to 90 mL/min/1.73 m^2^ (ΔeGFR −9 mL/min/1.73 m^2^, *P* < .01; Fig. 1A and 1B). In those who required >3 months for IGF-1 normalization, eGFR also declined significantly at POD14 (ΔeGFR −13 mL/min/1.73 m^2^, *P* = .04), with no further significant change between POD14 and 3 months postoperatively (*P* = .66; Fig. 1C and 1D).

### Postoperative renal changes by baseline eGFR

Based on a previously described protocol [5], we next stratified our patients according to whether they had baseline eGFR values of ≥90 mL/min/1.73 m^2^, and analyzed differences in postoperative eGFR decline.

**Figure 2:**
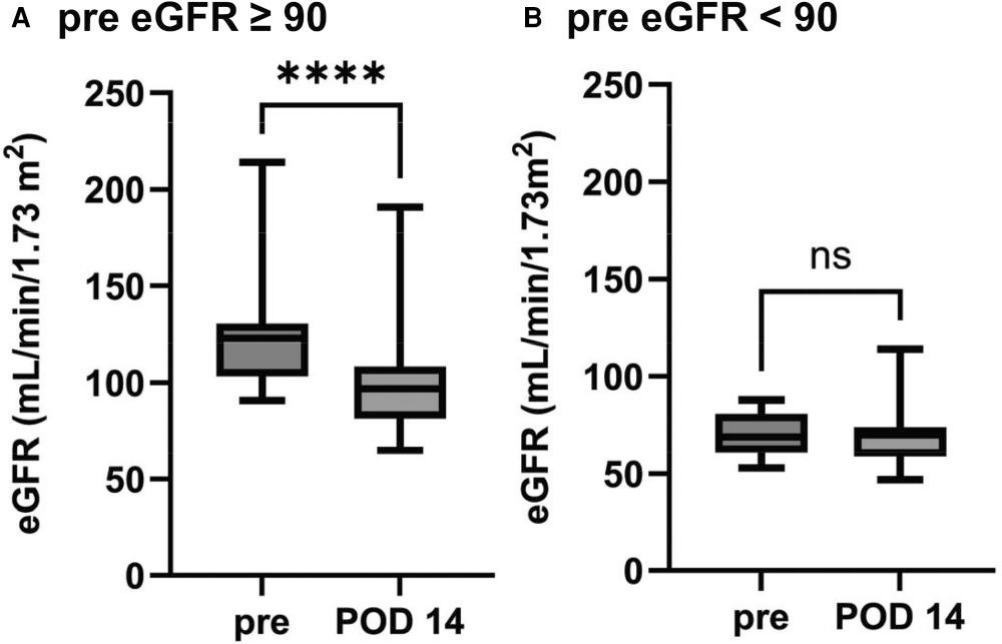
Early postoperative eGFR changes by baseline renal function. (A) The patients with baseline eGFR values of ≥90 mL/min/1.73 m^2^ (n = 33) showed a significant decline in eGFR between their preoperative and postoperative day 14 (POD14) values. (B) The patients with baseline eGFR values of <90 mL/min/1.73 m^2^ (n = 11) showed no significant change between their preoperative and POD14 values. Data are shown as medians ± interquartile ranges. ****P < .0001; ns, not significant.

**Figure 3:**
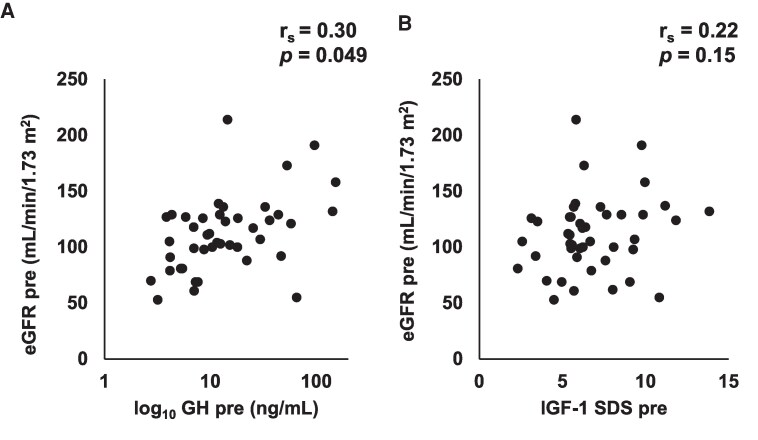
Correlations between baseline eGFR and preoperative GH or IGF-1 SDS. Preoperative GH correlated positively with baseline eGFR, whereas IGF-1 SDS showed no significant correlation.

The patients with preoperative eGFR values of ≥90 mL/min/1.73 m^2^ (n = 33) showed a significant decline in eGFR (ΔeGFR −27 mL/min/1.73 m^2^, *P* < .0001; Fig. 2A). Conversely, those with preoperative eGFR values of <90 mL/min/1.73 m^2^ (n = 11) showed no significant postoperative eGFR change (ΔeGFR −3 mL/min/1.73 m^2^, *P* = .38; Fig. 2B). Preoperative GH correlated positively with baseline eGFR, whereas IGF-1 SDS showed no significant correlation (Fig. 3). The distribution of chronic kidney disease (CKD) stages at each time point is summarized in Table S1 [14]. The proportion of patients with reduced renal function (eGFR < 60 mL/min/1.73 m^2^, corresponding to CKD stage G3a or below) remained low during the early postoperative period, and no patients progressed to CKD stage G3b or higher.

### Influence of age and sex on postoperative renal changes

Multivariate linear regression using preoperative eGFR as the dependent variable showed that age (β = −.46, *P* < .001) and female sex (β = −.28, *P* = .02) were significantly associated with preoperative eGFR, and were therefore used for subsequent stratified analyses.

**Figure 4:**
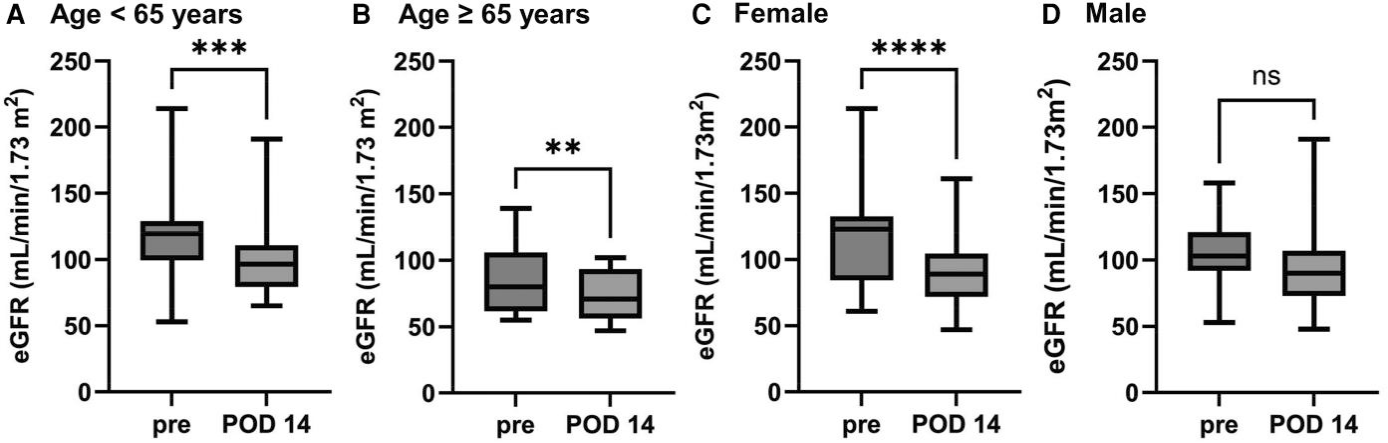
Early postoperative eGFR changes by age and sex. (A) The patients aged < 65 years (n = 34), (B) the patients aged ≥65 years (n = 10), (C) females (n = 25), and (D) males (n = 19). Significant eGFR declines were observed in both younger and older patients, with smaller ΔeGFR values in the older group. A significant postoperative decrease was observed in females, but not in males. The difference in eGFR between the postoperative day 14 (POD14) value and the preoperative one was defined as the ΔeGFR (POD14 eGFR—preoperative eGFR). Data are shown as medians ± interquartile ranges. **P < .01; ***P < .001; ****P < .0001; ns, not significant.

When stratified by age, younger patients (<65 years, n = 34) had significantly higher preoperative eGFR (Table 1) and a significant decline in eGFR (ΔeGFR −22 mL/min/1.73 m^2^, *P* < .001; Fig. 4A). Older patients (≥65 years, n = 10) also demonstrated a significant but more modest decline in eGFR (ΔeGFR −9 mL/min/1.73 m^2^, *P* = .002; Fig. 4B). Among older patients, 3 patients exhibited declines in eGFR to below 60 mL/min/1.73 m^2^ at POD14 despite having preoperative values above this threshold.

When stratified by sex, a significant postoperative eGFR decline was observed in females (*P* < .0001; Fig. 4C), but not in males (*P* = .11; Fig. 4D).

### Factors associated with postoperative eGFR change

We next identified which factors were associated with the magnitude of postoperative renal function change. To explore these factors, univariate correlation analyses and a multivariate regression analysis were performed, with ΔeGFR as the dependent variable and age, sex, BMI, ΔGH, ΔIGF-1 SDS, and the use of RASi or diuretics as covariates.

**Figure 5:**
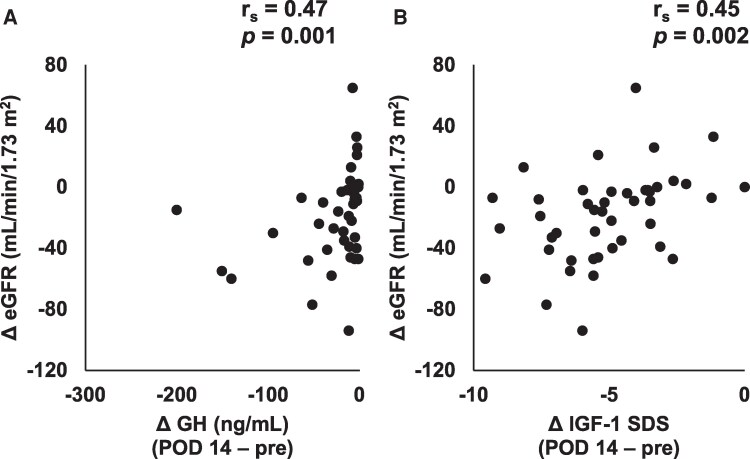
Correlations of GH and IGF-1 with postoperative eGFR change. (A) Postoperative GH change (ΔGH) correlated positively with ΔeGFR. (B) Postoperative IGF-1 SDS change (ΔIGF-1 SDS) also correlated positively with ΔeGFR. ΔeGFR, ΔGH, and ΔIGF-1 SDS were defined as the respective differences in their postoperative day 14 (POD14) values and their preoperative ones (POD14 value—preoperative value).

In the univariate correlation analyses, ΔeGFR positively correlated with both ΔGH and ΔIGF-1 SDS (Fig. 5). The multivariate regression analysis revealed that female sex and ΔIGF-1 SDS were significantly associated with postoperative ΔeGFR, whereas age, BMI, ΔGH, and the use of RASi or diuretics were not significant (Table 2). Because ΔeGFR and ΔIGF-1 SDS were defined as POD14—preoperative values, decreases were represented as negative values. Accordingly, the positive coefficient for ΔIGF-1 SDS indicated that greater postoperative reductions in IGF-1 were associated with larger eGFR declines, whereas the negative coefficient for female sex suggested that females had a greater likelihood of postoperative eGFR declines vs males.

**Table 2:** Multivariate regression analysis of the factors associated with ΔeGFR

Variable	Coefficient (β)	95% CI	*P* value
Age	.08	−0.57 to 0.91	.64
Female (vs male)	−.41	−20.10 to −3.89	.005
BMI	−.28	−5.42 to 0.57	.11
The use of RASi or diuretics	−.12	−12.5 to 5.50	.43
ΔGH (POD14—preoperative)	.01	−0.24 to 0.24	.97
ΔIGF-1 SDS (POD14—preoperative)	.36	0.74 to 9.16	.02

Values are expressed as regression coefficients (β) with 95% confidence intervals (CI) and *P* values.

ΔeGFR, ΔGH, and ΔIGF-1 SDS were defined as the respective differences between their postoperative day 14 (POD14) and preoperative values (POD14—preoperative values).

Abbreviations: BMI, body mass index; eGFR, estimated glomerular filtration rate; GH, growth hormone; IGF-1, insulin-like growth factor-1; RASi, renin–angiotensin system inhibitors; SDS, standard deviation score.

In the exploratory percentage-change analysis, %eGFR was significantly correlated with both %GH and %IGF-1 in univariate analyses (Fig. S1 [14]). However, in multivariate regression analysis, %eGFR was not significantly associated with either %GH or %IGF-1. In contrast, female sex and BMI were significantly associated with %eGFR (Table S2 [14]).

No significant differences in ΔCK were observed between sexes (*P* = .33) or between younger vs older patients (*P* = .90).

### Exploratory analysis of long-term renal function

To explore changes in long-term renal function, an additional exploratory analysis was performed on patients with follow-up data available up to 3 years after surgery (n = 18) (Fig. S2 [14]). In this subset, eGFR showed a significant decline at 1 year, which persisted at 3 years postoperatively. The median ΔeGFR was −28 mL/min/1.73 m^2^ at 1 year and −29 mL/min/1.73 m^2^ at 3 years postoperatively. At 3 years, only 2 of the 18 patients had eGFR values below 60 mL/min/1.73 m^2^. One patient exhibited a marked decline in eGFR from 79 mL/min/1.73 m^2^ to 32 mL/min/1.73 m^2^ at 3 years postoperatively. This patient was 65 years old at diagnosis and had a history of hypertension and diabetes, with HbA1c of 5.8%. Another patient had eGFR of 70 mL/min/1.73 m^2^ preoperatively, which declined to 52 mL/min/1.73 m^2^ at 3 years. This patient was 63 years old at diagnosis and also had diabetes, with an HbA1c level of 7.2%.

## Discussion

This study demonstrated that eGFR significantly declined within 14 days after trans-sphenoidal surgery in patients with acromegaly—particularly in younger patients, females, and those with high baseline eGFR values. Importantly, this study focused on the very early postoperative period, a clinically vulnerable phase characterized by dynamic hemodynamic changes [6], while accounting for patient-specific factors such as age and sex.

GH exerts direct effects on the kidneys through GHR-mediated pathways, with many of its renal actions being mediated indirectly via systemic or locally produced IGF-1. These mechanisms together account for approximately a 15% increase in eGFR in patients with acromegaly compared with healthy individuals [3]. Mechanistically, this hyperfiltration is attributed to: (1) nitric oxide (NO) mediated dilation of both afferent and efferent arterioles, which increases GFR and renal plasma flow; (2) upregulation of sodium phosphate cotransporters (Na-Pi2a/2c) in the proximal tubules; and (3) increased expression of epithelial sodium channels (ENaC) in the distal nephron [3]. Morphological renal hypertrophy has also been reported [15]. This change is reversible in many patients after pituitary tumor resection; however, this is not always the case.

Fujio et al [5] previously reported that significant eGFR reductions at 3 months postoperatively occurred only in patients with baseline eGFR values of ≥90 mL/min/1.73 m^2^. Consistent with their findings, our results demonstrated that eGFR had already declined significantly by POD14 only in patients with high preoperative eGFR values of ≥90 mL/min/1.73 m^2^. Notably, even in patients who required > 3 months postoperatively to achieve IGF-1 normalization, eGFR had already declined significantly at POD14. These findings suggest that early renal changes likely represent reversible hemodynamic adaptation following rapid reductions in GH/IGF-1. In this study, patients with preoperative eGFR values of <90 mL/min/1.73 m^2^ showed no significant postoperative changes. In CKD, impaired GH signaling and IGF-1 bioavailability may attenuate hyperfiltration responses [3, 16], potentially explaining the absence of early eGFR decline in this subgroup.

Our multivariate regression analysis revealed that age and sex were significantly associated with baseline eGFR. Older patients had lower baseline eGFR, possibly owing to increased renal vascular resistance and reduced hyperfiltration capacity [17]. In addition, the higher use of RASi among older patients may have attenuated GH/IGF-1-mediated renal effects.

Our stratified analysis showed that significant postoperative declines in eGFR occurred in both younger and older patients. Older patients generally have reduced renal reserves due to nephron loss and glomerulosclerosis [18], and advanced age is known as a risk factor for postoperative AKI [7]. However, in our study, the magnitude of eGFR decline was smaller in older patients, and no abrupt or clinically significant deterioration was observed. These findings suggest that early postoperative renal changes remain modest in older patients without pre-existing CKD.

Nevertheless, 3 older patients experienced declines in eGFR to below 60 mL/min/1.73 m^2^ in the early postoperative period despite having preoperative values above this threshold, all of whom had diabetes and hypertension. Furthermore, in an exploratory analysis of patients with long-term follow-up, eGFR declined significantly up to 3 years after surgery. Notably, only a small proportion of patients developed eGFR values below 60 mL/min/1.73 m^2^, 1 older patient with both diabetes and hypertension showed a decline in eGFR to 32 mL/min/1.73 m^2^ at 3 years. Previous studies have shown that age and diabetes are major predictors of CKD development in acromegaly [19], as well as an increased risk of end-stage kidney disease [20]. Taken together, although early postoperative eGFR decline is generally limited, older patients with metabolic comorbidities may be at risk for clinically relevant renal impairment and should be carefully monitored.

Our univariate correlation analysis revealed significant correlations between ΔeGFR and both ΔGH and ΔIGF-1 SDS. In multivariate analysis, ΔIGF-1 SDS, but not ΔGH, was independently associated with ΔeGFR. This finding may reflect mechanistic differences between GH and IGF-1 in renal physiology. While many renal effects of GH are mediated indirectly through hepatic and local IGF-1 production, IGF-1 directly stimulates proximal tubular and endothelial cells via IGF-1R, promoting sodium reabsorption and increasing renal blood flow [3]. Exploratory analyses using percentage changes showed that neither %GH nor %IGF-1 remained independently associated with %eGFR in multivariate analysis, in which female sex and BMI were the only significant factors. These findings suggest that simple percentage changes in hormonal levels may not fully capture the relationship between hormonal improvement and renal function. Given the limited sample size and the number of variables included, these results should be interpreted with caution.

Sex-based differences were also identified, with female sex being independently associated with ΔeGFR. Estrogen may enhance renal vascular responsiveness through nitric oxide-mediated pathways and modulation of the renin–angiotensin system [21, 22], potentially contributing to greater eGFR decline in females. This finding may support the concept of sex-specific hormonal regulation of renal function, although further mechanistic studies are needed.

This study has several limitations. First, it was a single-center, retrospective study with a limited sample size, which reduced the statistical power for the analyses. Second, eGFR was estimated using a sCr–based equation, which may be influenced by muscle mass [12]. Although CK was evaluated as an indirect indicator of muscle mass [13], it is not a direct measure of muscle mass. While postoperative CK remained stable across age and sex, the potential influence of unmeasured changes in muscle mass cannot be fully excluded. Future studies should consider introducing direct assessments, such as body composition analysis, or utilizing markers independent of muscle mass, such as cystatin C, to further validate these findings. Third, since this study focused on early postoperative renal changes, long-term renal function was only assessed in a subset of patients. Therefore, the clinical implications of long-term prognosis should be interpreted with caution. Additionally, since direct measurements of renal hemodynamics were not performed, interpreting the observed decline in eGFR as an improvement in filtration function is speculative.

Despite these limitations, to our knowledge, this study is the first to investigate changes in renal function following surgical treatment in patients with acromegaly during the early postoperative period, when hemodynamic status is highly variable. Notably, this study also includes a focused analysis of older patients, a group that has been underrepresented in previous research. Considering preoperative renal function, age, and sex, our findings may help inform postoperative monitoring and risk stratification in this population.

## Conclusion

Our findings suggest that excess GH/IGF-1 represents a key driver of glomerular hyperfiltration in acromegaly, and that surgical remission leads to rapid normalization of renal hemodynamics, particularly in younger patients, females, and those with higher baseline eGFR values. Although postoperative declines in eGFR were generally modest, patients with advanced age and metabolic comorbidities such as diabetes and hypertension may be more vulnerable to clinically relevant declines and warrant careful monitoring. Female sex and ΔIGF-1 SDS were independently associated with early postoperative eGFR change, suggesting that renal responsiveness to GH/IGF-1 normalization is at least partially sex-specific. Future longitudinal studies are warranted to determine whether these early post-treatment changes translate into improved long-term renal outcomes.

## Data Availability

Some or all datasets generated during and/or analyzed during the current study are not publicly available but are available from the corresponding author on reasonable request.

## References

[1] Melmed S . Acromegaly pathogenesis and treatment. J Clin Invest. 2009;119(11):3189‐3202. 19884662 10.1172/JCI39375 PMC2769196

[2] Kamenický P, Mazziotti G, Lombès M, Giustina A, Chanson P. Growth hormone, insulin-like growth factor-1, and the kidney: pathophysiological and clinical implications. Endocr Rev. 2014;35(2):234‐281. 24423979 10.1210/er.2013-1071

[3] Haffner D, Grund A, Leifheit-Nestler M. Renal effects of growth hormone in health and in kidney disease. Pediatric Nephrol. 2021;36(8):2511‐2530. 10.1007/s00467-021-05097-6 PMC8260426 34143299

[4] Kamenicky P, Blanchard A, Frank M, et al Body fluid expansion in acromegaly is related to enhanced epithelial sodium channel (ENaC) activity. J Clin Endocrinol Metab. 2011;96(7):2127‐2135. 21508131 10.1210/jc.2011-0078

[5] Fujio S, Takano K, Arimura H, et al Treatable glomerular hyperfiltration in patients with active acromegaly. Eur J Endocrinol. 2016;175(4):325‐333. 27440194 10.1530/EJE-16-0242

[6] Kheterpal S, Tremper KK, Heung M, et al Development and validation of an acute kidney injury risk index for patients undergoing general surgery: results from a national data set. Anesthesiology. 2009;110(3):505‐515. 19212261 10.1097/ALN.0b013e3181979440

[7] Zhang M, Zeng J, Ge Y, et al Risk factors and prognosis of post-surgical acute kidney injury in elderly patients based on the MIMIC-IV database. Eur J Med Res. 2025;30(1):491. 40537854 10.1186/s40001-025-02762-6 PMC12178035

[8] Neugarten J, Golestaneh L. Female sex reduces the risk of hospital-associated acute kidney injury: a meta-analysis. BMC Nephrol. 2018;19(1):314. 30409132 10.1186/s12882-018-1122-z PMC6225636

[9] Isojima T, Shimatsu A, Yokoya S, et al Standardized centile curves and reference intervals of serum insulin-like growth factor-I (IGF-I) levels in a normal Japanese population using the LMS method. Endocr J. 2012;59(9):771‐780. 22673406 10.1507/endocrj.ej12-0110

[10] Brue T, Rahabi H, Barry A, et al Position statement on the diagnosis and management of acromegaly: the French National Diagnosis and Treatment Protocol (NDTP). Ann Endocrinol (Paris). 2023;84(6):697‐710. 37579837 10.1016/j.ando.2023.08.003

[11] Guo X, Zhang R, Zhang D, et al Determinants of immediate and long-term remission after initial transsphenoidal surgery for acromegaly and outcome patterns during follow-up: a longitudinal study on 659 patients. J Neurosurg. 2022;137(3):618‐628. 35171834 10.3171/2021.11.JNS212137

[12] Baxmann AC, Ahmed MS, Marques NC, et al Influence of muscle mass and physical activity on serum and urinary creatinine and serum cystatin C. Clin J Am Soc Nephrol. 2008;3(2):348‐354. 18235143 10.2215/CJN.02870707 PMC2390952

[13] Chen Z, Laurentius T, Fait Y, et al Associations of Serum CXCL12alpha and CK levels with skeletal muscle mass in older adults. J Clin Med. 2023;12(11):3800. 37297995 10.3390/jcm12113800 PMC10253690

[14] Tomiyama K, Fukuda I, Okazaki-Hada M, et al Data from: Supplemental Data for: “Preoperative eGFR, age, and sex predict early postoperative renal changes in acromegaly”. *Figshare*. 2026. Deposited June 1, 2026. Accessed June 1, 2026. 10.6084/m9.figshare.32526660 PMC13265133 42299319

[15] Auriemma RS, Galdiero M, De Martino MC, et al The kidney in acromegaly: renal structure and function in patients with acromegaly during active disease and 1 year after disease remission. Eur J Endocrinol. 2010;162(6):1035‐1042. 20356933 10.1530/EJE-10-0007

[16] Rabkin R, Sun DF, Chen Y, Tan J, Schaefer F. Growth hormone resistance in uremia, a role for impaired JAK/STAT signaling. Pediatric Nephrol. 2005;20(3):313‐318. 10.1007/s00467-004-1713-8 15692835

[17] Premaratne E, Macisaac RJ, Tsalamandris C, Panagiotopoulos S, Smith T, Jerums G. Renal hyperfiltration in type 2 diabetes: effect of age-related decline in glomerular filtration rate. Diabetologia. 2005;48(12):2486‐2493. 16261309 10.1007/s00125-005-0002-9

[18] Denic A, Glassock RJ, Rule AD. Structural and functional changes with the aging kidney. Adv Chronic Kidney Dis. 2016;23(1):19‐28. 26709059 10.1053/j.ackd.2015.08.004 PMC4693148

[19] Castagna G, Ippolito S, Cassibba S, et al Kidney function in acromegaly: evidence from a long-term observational study. Pituitary. 2025;28(3):56. 40329088 10.1007/s11102-025-01520-5 PMC12055623

[20] Hong S, Kim KS, Han K, Park CY. A cohort study found a high risk of end-stage kidney disease associated with acromegaly. Kidney Int. 2023;104(4):820‐827. 37490954 10.1016/j.kint.2023.06.037

[21] Veser C, Carlier A, Dubois V, Mihaila SM, Swapnasrita S. Embracing sex-specific differences in engineered kidney models for enhanced biological understanding of kidney function. Biol Sex Differ. 2024;15(1):99. 39623463 10.1186/s13293-024-00662-8 PMC11613810

[22] Baylis C . Sexual dimorphism, the aging kidney, and involvement of nitric oxide deficiency. Semin Nephrol. 2009;29(6):569‐578. 20006788 10.1016/j.semnephrol.2009.07.003 PMC2796249

